# Pupil response to social-emotional material is associated with rumination and depressive symptoms in adults with autism spectrum disorder

**DOI:** 10.1371/journal.pone.0200340

**Published:** 2018-08-07

**Authors:** Katherine O. Gotham, Greg J. Siegle, Gloria T. Han, Andrew J. Tomarken, Rachel N. Crist, David M. Simon, James W. Bodfish

**Affiliations:** 1 Department of Psychiatry and Behavioral Sciences, Vanderbilt University Medical Center, Nashville, Tennessee, United States of America; 2 Department of Psychiatry, University of Pittsburgh Medical Center, Department of Psychology, University of Pittsburgh, Pittsburgh, Pennsylvania, United States of America; 3 Department of Psychology, Vanderbilt University, Nashville, Tennessee, United States of America; 4 Neuroscience Graduate Program, Vanderbilt Brain Institute, Vanderbilt University, Nashville, Tennessee, United States of America; 5 Department of Hearing and Speech Sciences, Vanderbilt University Medical Center, Nashville, Tennessee, United States of America; Harvard Medical School, UNITED STATES

## Abstract

**Background:**

Autism spectrum disorder (ASD) is marked by repetitive thinking and high rates of depression. Understanding the extent to which repetitive negative thinking in ASD reflects autistic stereotypy versus general depressive thinking patterns (e.g., rumination) could help guide treatment research to improve emotional health in ASD. We compared associations between rumination, depressive symptoms, and pupil response to social-emotional material in adults with ASD and typically developing (TD) adults with and without depression.

**Methods:**

N = 53 verbally fluent young adults were recruited to three cohorts: ASD, n = 21; TD-depressed, n = 13; never-depressed TD-controls, n = 19. Participants completed Ruminative Response Scale and Beck Depression Inventory self-reports and a passive-viewing task employing emotionally-expressive faces, during which pupillary motility was assessed to quantify cognitive-affective load. Main and interactive effects of cohort, emotion condition, and time on pupil amplitude were tested via a linear mixed effects analysis of variance using restricted maximum likelihood estimation. Similar procedures were used to test for effects of rumination and depressive symptoms on pupil amplitude over time within ASD.

**Results:**

Responsive pupil dilation in the ASD cohort tended to be significantly lower than TD-depressed initially but increased to comparable levels by trial end. When viewing sad faces, individuals with ASD who had higher depression scores resembled TD-depressed participants’ faster, larger, and sustained pupil response. Within ASD, depressive symptoms uniquely predicted early pupil response to sad faces, while rumination and depression scores each independently predicted sustained pupil response.

**Conclusions:**

People with elevated depressive symptoms appear to have faster and greater increases in pupil-indexed neural activation following sad stimuli, regardless of ASD status, suggesting the utility of conceptualizing rumination as depression-like in treatment. Ruminative processes may increase more slowly in ASD, suggesting the potential utility of interventions that decrease reactions before they are uncontrollable. Findings also reinforce the importance of testing for effects of internalizing variables in broader ASD research.

## Introduction

Repetitive cognition and behaviors comprise a core symptom domain of autism spectrum disorder (ASD). When observed clinically, repetitive focus within ASD varies widely by topic: from strong special interests that appear rewarding, to unusual focus on more neutral content (e.g., a grocery list), to negative or self-critical foci often associated with negative affect (e.g., a job failure; thoughts of “I never fit in”). Passive, repetitive negative thinking, or rumination, is robustly associated with the onset of depressive disorders in the typically developing (TD) population [1, 2]. Rates of depression are notably high in adolescents and adults with ASD [3, 4]–estimates of 50–75% lifetime occurrence of a major depressive episode are reported even in young adult samples with ASD assessed with standardized interviews—and ruminative thinking has been shown to prospectively predict increased depressive symptoms in children with ASD [5].

As repetitive negative thinking likely contributes to depressed mood in ASD [5–8], intermediate processes in this pathway may guide treatment decisions in this population. Specifically, the extent to which rumination in autism is “depression-like”–i.e., similar to rumination in TD-depressed individuals–could suggest whether to look first to therapeutic strategies founded in depression science or those specific to autism. If repetitive negative thinking in ASD has *different* neural signatures than it does in TD individuals, treatments may best target autism-like cognitive perseveration more broadly. Alternatively, rumination and depression could have *similar* intermediate and interactive pathways in ASD as they do in the general population, suggesting that existing interventions [9, 10] may be applicable to these features within autism. In the current study, we used psychophysiological measures to examine the extent to which sustained cognitive-affective load, a mechanism implicated in both rumination and depression, (1) looked similar or different between people with ASD and TD-depressed individuals, and (2) varied with self-reported rumination and depressive symptoms in people with ASD.

Our proxy for cognitive-affective load was pupil dilation in response to emotional information. In typically developing depressed and anxiety-disordered samples, pupil dilation, like other indices of neural reactivity [11, 12], has been shown to endure after offset of emotional stimuli [13–15]. Pupil reactivity reflects sympathetic and parasympathetic nervous system activity, and covaries with changes in other physiological measures such as skin conductance [16, 17]. Pupil dilation in response to cognitive tasks is associated with brain activity primarily in "task-network" regions, such as the dorsolateral prefrontal cortex [18, 19], and reliably measures cognitive load (e.g., solving a harder versus easier math problem). Increased dilation also is elicted by both pleasant and unpleasant stimuli compared to neutral, and has been associated with heightened attention and sympathetic drive [16]. Pupil methods have been used successfully in the ASD population to explore neural reactivity to social-emotional stimuli [20–22].

Pupillometry offers the temporal resolution necessary to measure both immediate and sustained processing of stimuli. Depressed adults display greater *sustained* pupil dilation (within 9-second trials and an information processing task immediately following) in response to dysphoric stimuli than non-depressed individuals: This was particularly apparent in response to personally-generated words that participants reported thinking about when “upset, down, or depressed,” and the effect was moderately correlated with self-reported rumination [13]. Subsequent studies similarly report greater pupil dilation to dysphoric stimuli for individuals characterized by depression and greater ruminative style [23, 24]. However, in a larger, transdiagnostic sample [14], sustained pupil dilation was associated with affective but not cognitive-ruminative features of depression. In the present study, we aim to see if pupil response course to sad stimuli in a young adult ASD sample resembles that of a TD-depressed comparison group and replicates these previous findings from the depression literature. We also test how this pupil response is differentially associated over time with self-reported rumination and depressive symptoms within ASD.

We hypothesize that:

1. As ASD was the only cohort allowed to vary on depression status, both rumination and depression self-report scores of adults with ASD will fall between TD-depressed and never-depressed adults, who will have high and low group means, respectively. Repetitive behaviors traditionally associated with autism will be significantly higher in the ASD group than both TD comparison groups.
2. TD-depressed will have high and sustained pupil dilation to emotionally-relevant stimuli, and TD-controls will have lower and less sustained neural response. We have no prediction for general pupil response to emotion in adult ASD, given the heterogeneous levels of depressive symptoms in this population, as well as its potential anomalies in social-emotional information processing [25].
3. Higher levels of self-reported depressive symptoms and rumination within ASD will each be associated with greater pupil dilation to *sad* stimuli over time. If confirmed, this will provide novel evidence that increased cognitive-affective load in response to dysphoric stimuli marks rumination and depression within ASD as it does in the depressed population. The other emotion conditions will also be tested on an exploratory basis with no specific hypotheses.
4. Within ASD, rumination will be uniquely associated with sustained (i.e., late-in-trial) dilation to sad stimuli. Given previously-reported shared variance between rumination and depressive symptoms [26], we will test for the contribution of each predictor controlling for the other, on both early and sustained pupil response to sad stimuli.

Findings are intended to suggest to what degree repetitive negative thinking in ASD should be approached as depression-like versus population-unique, thus informing intervention decisions and research directions.

## Methods and materials

This research was performed in accordance with the Declaration of Helsinki. All procedures described herein received ethical review and approval from the Institutional Review Board of Vanderbilt University Medical Center (Behavioral Sciences Committee. Approval Number IRB# 141335). All participants were 18 years of age or older and served as their own legal representatives; we assessed capacity to provide informed consent for all participants, and obtained informed consent in writing from all participants, with consent forms approved by the board named above.

### Participants & procedures

A total of 53 participants aged 18–35 years were recruited from three diagnostic cohorts: Those with an autism spectrum disorder (ASD, n = 21), typically developing adults with a current depressive disorder (TD-depressed, n = 13), or typically developing comparisons with no history of an ASD or clinically significant depression or anxiety (TD-controls, n = 19). Participants were recruited from national and local resources, including ResearchMatch, a state autism association, core recruitment services at a Eunice Kennedy Shriver National Institute of Child Health and Human Development Center for Human Development, and patient enrollment at a large academic medical center in the mid-Southern United States. Eligibility criteria included verbal IQ> = 80; verbal fluency per Autism Diagnostic Observation Schedule, 2^nd^ edition (ADOS-2) [27] module selection criteria; reading level > = 5^th^ grade; 20/20 vision at 80 cm on the Snellen eye chart; and no history or concerns of psychotic or bipolar disorders, current substance use disorders, or uncorrected vision problems or ocular abnormalities. Participants in the clinical cohorts had previous diagnoses of ASD or depressive disorder, respectively. Table 1 provides demographic information by cohort.

**Table 1:** Sample demographics and self-report descriptives.

Mean(SD) Range; α	TD-controls	ASD	TD-dep	Group differences^a^
N	19	21	13	
Age in Years	26.6(4.5) 21–35	22.4(4.6) 18–33	23.9(3.3) 19–32	F(2, 32.52) = 4.24, p = .023; TD-con>ASD, p = .003
Verbal IQ	114.4(14.3) 90–147	101(10.9) 81–120	115(8.4) 98–130	F(2, 32.01) = 10.06, p < .001; TD-con>ASD, p < .001 TD-dep>ASD, p = .001
Nonverbal IQ	107.9(15.1) 79–136	106.6(15.2) 71–141	110.3(9.7) 89–126	n.s.
Gender (% F/Other)	56%	19%	54 / 8%	χ^2^(1) = 6.4, p = .01(ASD<TD-con) χ^2^(1) = 6.9, p = .03(ASD<TD-dep)
Non-White	21%	5%	8%	n.s.
Highest Education (% HS or Less)	0	32%	0	χ^2^(2) = 11.45, p = .003 ASD<TD-con,TD-dep
BDI-II	2.0(2.1) 0–8; α = .66	11.5(10.0) 0–29; α = .90	24.5(6.3) 17–34; α = .71	F(2, 21.96) = 81.12, p < .001; TD-con<ASD<TD-dep (all p < .001)
RRS	30.6(8.0) 23–53; α = .91	42.3(13.4) 23–74; α = .93	53.3(7.0) 39–65; α = .69	F(2, 30.92) = 34.44, p < .001; TD-con<ASD (p = .003), TD-con<TD-dep (p < .001), ASD<TD-dep (p < .001)
RBS-R Total	3.7(5.0) 0–15	21.4(23.4) 0–79	12.1(15.0) 0–56	F(2, 21.08) = 6.94, p = .005; TD-con<ASD, p = .001
SRS-RRB T-score	43.2(3.7) 40–57	67.3(12.2) 50–87	55.1(10.6) 43–80	F(2, 22.51) = 40.73, p < .001; TD-con<ASD (p < .001), TD-con<TD-dep (p = .001), ASD>TD-dep (p < .001)
IS Intensity Total	8.1(1.7) 5–11	11.9(3.8) 7–20	10.0(1.8) 8–14	F(2, 28.45) = 9.61, p < .001; TD-con<ASD (p < .001)

^a^ Group differences reported only when Fisher’s LSD or chi-square tests of association significant at p < .05. See Supplementary Materials (S1 File) for empirically-based discussion of why observed group differences on demographic variables likely would not affect the current results, and S1 Table for key variable means by gender.

*Note*. TD-con = typically developing control adults with no history of depression or anxiety; ASD = adults with autism spectrum disorder; TD-dep = typically developing adults with current depressive disorders; BDI-II = Beck Depression Inventory, 2^nd^ edition; RRS = Ruminative Response Scale; RBS-R Total = Repetitive Behavior Scale-Revised overall total score; SRS-RRB T-score = Social Responsiveness Scale, 2^nd^ edition, Restricted Repetitive Behavior subscale T-score; IS Intensity Total = Interests Scale overall “Intensity” score.

Group differences in gender increase generalizability to the populations under comparison: From base rates of both disorders, we would expect to have more men represented in the ASD group and more women in the depressed cohort [28, 29]. From all indications in the literature, rumination is more prevalent in women than in men (in many samples, this mediates the gender effect in which more women than men become depressed [1]), however, the *mechanism* is not thought to function differently across men and women, such that, when rumination is present in males, it also tends to be associated with depression [1, 30]. We were not overly concerned about sex effects in this study, because even though the ASD group is significantly skewed toward males, it also had significantly higher average scores on measures of depression and rumination (see Table 1), which is opposite the direction of an expected sex bias. S1 Table presents gender comparisons. See Supplementary Materials (S1 File) for more empirically-based discussion of why observed group differences on demographic variables likely would not affect the current results.

Procedures were approved by the Institutional Review Board of Vanderbilt University Medical Center, and included a telephone screening followed by 1–2 data collection lab visits. The ADOS-2 was administered to all participants in the ASD cohort to confirm diagnosis, as well as to any participants who exceeded clinical cut-offs on the Social Responsiveness Scale (SRS-2) [31, 32] or Autism Spectrum Quotient (AQ) [33]. The Structured Clinical Interview for DSM Disorders (SCID-5) [34] depression module and the Mini International Neuropsychiatric Interview (MINI 5.0) [35] were administered to all participants to confirm diagnosis and/or assess emotional health history. In the ASD cohort, 76% (16/21) met criteria for lifetime depressive disorders (n = 3 current), and 81% (17/21) had a history of anxiety disorder(s). In the TD-depressed group, all had current Major Depressive Disorder or Persistent Depressive Disorder, and 85% (11/13) had a history of anxiety disorder(s). See Supplementary Materials (S1 File) for additional procedures.

Self-report measures included the Beck Depression Inventory (BDI-II) [36] and the Ruminative Response Scale (RRS) [37], which are both commonly used, well-validated instruments that have been applied to adult ASD samples [6, 38]. Restricted, repetitive behaviors and strong fixed interests commonly associated with ASD were assessed with the Repetitive Behavior Scale-Revised (RBS-R) [39], the RRB subscale of the SRS-2, and the “Intensity” subscale of the Interests Scale (IS) [40]. Table 1 presents descriptive and reliability statistics; S2–S5 Tables provide correlations.

### Emotional faces task description

**Fig 1:**
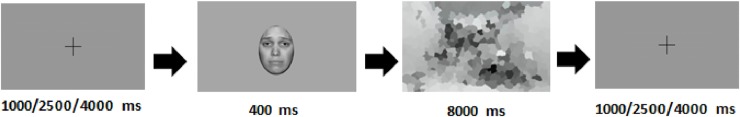
Emotional faces passive viewing task diagram. In the position of the second slide above, images were randomized across 80 trials, in which 4 expressions (Happy, Sad, Angry, Neutral) from 10 female and 10 male actors from the NimStim set [41] were each presented one time. The same mask (third slide above) was presented on each trial and was matched on luminance and low-level visual salience components to the face stimuli.

See Fig 1 for visual depiction of this passive-viewing task in which emotionally-salient faces were briefly presented, then followed by a mask for 8 seconds. A fixation cross first was displayed for 1, 2.5, or 4 seconds (randomly jittered across trials to avoid anticipatory responses and promote continued attention), followed by a single image of an emotionally-salient face for 400 milliseconds. Face images from the NimStim set [41] were cropped to remove hair and non-facial features, and were presented in random order across 20 actors (10 women, 10 men), each performing four expressions: Happy, Sad, Angry, and Neutral. In each of the 80 trials, the face image was followed by a scrambled mask shown for 8 seconds. We planned to analyze pupil magnitude during this longer-running neutral stimulus (the scrambled mask), in order to pull for sustained response to a priming emotional stimulus, while avoiding the blunting or habituation effects we might expect to see with prolonged viewing of the emotional stimulus itself.

All slides were shown in grayscale, and all were matched for mean luminance and low-level salient aspects, such as color and contrast, using the Spectral Visual Saliency Toolbox in Matlab [42, 43]. This matching protocol also applied to the scrambled mask, such that we would not expect random scanning patterns during the 8 second exploration of the mask to exert undue effects on pupil magnitude. See also Supplementary Materials (S1 File) for additional information.

### Pupil data acquisition & cleaning

Pupil tasks were displayed on a 24 inch/61cm (diagonal) monitor in a dimly lit, sparsely furnished room with few distractions. Participants were seated 55 cm from the screen. A Tobii X2-60 Hz eye-tracker recorded pupil data, allowing for non-invasive measurement and relatively free head movement. The eye-tracker collected data every 16.67 ms and was calibrated before each of three blocks of trials using a five-point calibration screen. All measurements refer to horizontal pupil diameter in millimeters. Pupil dilation at all time points within a single trial was individual-baseline corrected using the last 100 ms of the preceding fixation cross for each trial, with random presentation order helping to minimize potential effects of carryover from preceding trials. Linear interpolations replaced blinks throughout the dataset (see [44]). Trials comprised of over 50% blinks were excluded from further analysis; participants whose data were excluded for greater than 50% of the 80 possible trials were excluded from pupil analyses (n = 2 ASD, n = 1 TD-controls). See Supplementary Materials (S1 File) for further details.

Of note, we did not control for fixation on face stimuli in any of our analyses due to the near-complete uniformity of orientation to the faces within each diagnostic cohort (heatmaps can be recreated from eye gaze data in the Supplement or are available from the corresponding author on request). Given the task set-up, in which most viewing time is spent on a fixation cross and a scrambled mask that are standard across trials, the brief glimpses of faces were highly novel and thus salient; our data support the fact that participants oriented to them immediately and throughout their brief duration.

### Data analysis plan

Analyses were conducted using SAS Version 9.4 [45]. Following pupil cleaning procedures, 90% ASD, 100% TD-depressed, and 95% TD-controls remained non-missing. Psychometric data were complete except for one participant with ASD. The statistical procedures accommodated incomplete data in repeated measures or other multivariate contexts.

To identify significant group differences on self-report measures, we first computed Levene's test for homogeneity of variance across cohorts. Significant heterogeneity of variance was detected on BDI-II scores (F(2,48) = 18.10, p < .001), with a similar but non-significant trend on the RRS (F(2, 48) = 1.48, p = .237), so group differences on both measures were assessed using the Welch adjusted degree of freedom (WADF) robust alternative to one-way analysis of variance (ANOVA) [46].

We tested for the main and interactive effects of cohort, emotion condition (Happy/Sad/Angry/Neutral face), and time (resampled to 1 Hz yielding 8 samples representing the mean pupil diameter at each of seconds 1–8) on pupil amplitude using a mixed effects ANOVA specified in SAS PROC MIXED [47, 48]. Restricted maximum likelihood (REML) estimation was used, and general F-statistics and Kenward-Roger degrees of freedom were computed to test fixed effects. A Kronecker Product (KP) structure [49] on the residual covariance matrix was used to model within-subject dependency in pupil dilation across seconds and emotion conditions. An unrestricted covariance structure was used to model the covariance among a given participant’s pupil responses across emotion conditions, and an autoregressive lag-1 (AR1) structure was used to model the covariances across seconds. We used a similar analytic framework to test for the effect of rumination and depressive symptoms on pupil amplitude over time within ASD, with primary focus on sad stimuli.

Finally, we examined the unique versus joint effects of rumination and depressive symptoms on early and sustained pupil response to sad stimuli in ASD. We defined “early” pupil response as the average of baseline-corrected pupil magnitude in millimeters at seconds 2 and 3 (following the resolution of the light reflex in response to the change between face and mask stimuli), and “late” pupil response as the average across seconds 5–8. Informed by Treynor and colleagues [26], we used the RRS Brooding subscale for these analyses to minimize confounding and similar item content between our rumination and depression measures. To examine incremental variance associated with rumination or depression above and beyond the other variable, we first entered RRS-Brooding and BDI-II as sole explanatory variables of either early or late pupil averages (as separate models) in sequential multiple regression analyses; in the second step, both explanatory variables were included in the same model. The squared semi-partial correlation (*sr*^2^) between each predictor and pupil response was computed, indicating proportion of total variance of the dependent measure uniquely accounted for by a given predictor. Given that the shared (i.e., overlapping) influence of two predictors can be quantified as model R^*2*^– Σ^sr2^, we could quantify both the unique and shared components of variance for rumination and depressive symptoms.

## Results

### Cohort differences on self-report measures

The cohorts differed significantly on rumination and depressive symptoms as hypothesized (TD-controls < ASD < TD-depressed; see Table 1). A distinct pattern was observed on measures of repetitive behaviors traditionally associated with autism: TD-controls were significantly lower than ASD across instruments, but TD-depressed and ASD group averages were not consistently different.

### Cohort differences in pupil response over time to social-emotional stimuli

**Fig 2:**
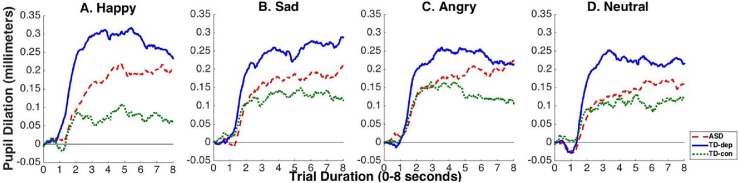
Averaged baseline-corrected pupil dilation across 8 second trials, by emotion condition and diagnostic cohort. The x-axes represent the averaged 8 second viewing of the scrambled mask (see Fig 1) following emotional face stimuli of the category labeled in each panel. ASD = adults with autism spectrum disorder; TD-dep = typically developing adults with current depressive disorders; TD-con = typically developing control adults with no history of depression or anxiety.

**Fig 3:**
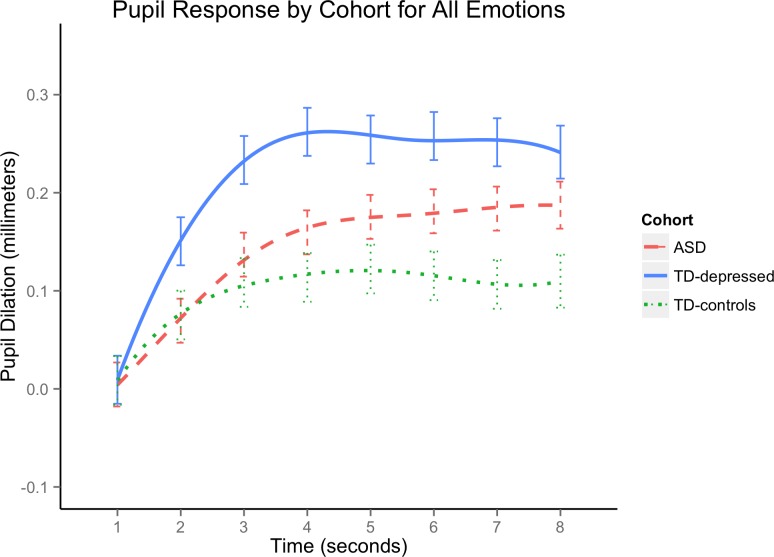
Pupil Response by Cohort for All Emotions. The x-axis represents the 8 second viewing of the scrambled mask (see Fig 1) following emotional face stimuli presentation. ASD = adults with autism spectrum disorder; TD-depressed = typically developing adults with current depressive disorders; TD-controls = typically developing comparison adults with no history of depression or anxiety. Because the functions appeared smooth, we generated plots using cubic splines with two knots. Significant differences tested at seconds 1 through 8 at Bonferroni-adjusted α = .05/8 = 0.00625 were: TD-controls<TD-depressed at seconds 2 through 8; ASD<TD-depressed at seconds 2 through 6, and TD-controls<ASD at seconds 6 through 8 (see Table 2 for details).

Fig 2 displays “raw” pupil data (i.e., cleaned and baseline-corrected data sampled at the original 60 Hz) averaged by cohort in each of the four emotion conditions. In data resampled to 1 Hz, yielding one averaged pupil diameter estimate for each of 8 seconds, the combination of a significant cohort X time interaction (F(14, 339) = 4.35, p < .0001) and non-significant cohort X emotion condition X time interaction (F(42, 856) = .81, p = .80) indicated that significant differences in pupil response over time by diagnostic cohorts were not driven by differences in emotion condition. Thus, for maximum interpretability, Fig 3 displays smoothed pupil curves averaged across all emotion conditions for each cohort (smoothing was achieved using cubic splines with two knots, a method used here only for graphical representation). Table 2 presents complementary statistics on cohort differences in pupil magnitude at each of the 8 seconds viewing the scrambled mask. Bonferroni-corrected pairwise comparisons confirmed that TD-depressed had significantly greater pupil response than both ASD and TD-controls in the early part of the trials following the light reflex. However, the ASD cohort increased in pupil magnitude to differ significantly from TD-controls by second 6 and beyond, and no longer differed significantly from TD-depressed at seconds 7 and 8.

**Table 2:** Between-cohort differences in pupil dilation to aggregated emotion conditions at seconds 1 through 8.

Mean (mm) [SD = .02 for all]	**Sec 1** F(2, 275) = .03, p = .975	**Sec 2** F(2, 275) = 6.35, p = .002	**Sec 3** F(2, 275) = 13.37, p < .0001	**Sec 4** F(2, 275) = 18.20, p < .0001
**TD-con < TD-dep**	TD-con: .009; TD-dep: .009 t(275) = -.03, p = .98	TD-con: 0.08; TD-dep: 0.15 *t(275) = -3*.*03*, *p =* .003*	TD-con: 0.11; TD-dep: 0.23 *t(275) = -5*.*03*, *p <* .0001*	TD-con: 0.114; TD-dep: 0.26 *t(275) = -5*.*98*, *p <* .0001*
**TD-con < ASD**	TD-con: .009; ASD: .004 t(275) = .18, p = .85	TD-con: 0.08; ASD: 0.07 t(275) = .26, p = .80	TD-con: 0.11; ASD: 0.14 t(275) = -1.27, p = .207	TD-con: 0.11; ASD: 0.16 t(275) = -2.05, p = .042
**ASD < TD-dep**	ASD: .004; TD-dep: .009 t(275) = -.20, p = .84	ASD: 0.07; TD-dep: 0.15 *t(275) = -3*.*30*, *p =* .001*	ASD: 0.14; TD-dep: 0.23 *t(275) = -3*.*93*, *p =* .0001*	ASD: 0.114; TD-dep: 0.262 *t(275) = -4*.*17*, *p <* .0001*
	**Sec 5** F(2, 275) = 14.15, p < .0001	**Sec 6** F(2, 275) = 16.52, p < .0001	**Sec 7** F(2, 275) = 17.38, p < .0001	**Sec 8** F(2, 275) = 14.67, p < .0001
**TD-con < TD-dep**	TD-con: 0.12; TD-dep: 0.25 *t(275) = -5*.*32*, *p <* .0001*	TD-con: 0.115; TD-dep: 0.26 *t(275) = -5*.*74*, *p <* .0001*	TD-con: 0.107; TD-dep: 0.25 *t(275) = -5*.*84*, *p <* .0001*	TD-con: 0.11; TD-dep: 0.24 *t(275) = -5*.*30*, *p <* .0001*
**TD-con < ASD**	TD-con: 0.12; ASD: 0.18 t(275) = -2.37, p = .019	TD-con: 0.115; ASD: 0.181 *t(275) = -2*.*93*, *p =* .0037*	TD-con: 0.107; ASD: 0.184 *t(275) = -3*.*45*, *p =* .0007*	TD-con: 0.11; ASD: 0.19 *t(275) = -3*.*45*, *p =* .0006*
**ASD < TD-dep**	ASD: 0.18; TD-dep: 0.25 *t(275) = -3*.*21*, *p =* .002*	ASD: 0.181; TD-dep: 0.258 *t(275) = -3*.*12*, *p =* .002*	ASD: 0.107; TD-dep: 0.252 t(275) = -2.75, p = .0063	ASD: 0.19; TD-dep: 0.24 t(275) = -2.20, p = .029

* Significant differences at Bonferroni-adjusted α = .05/6 = 0.00625.

ASD = adults with autism spectrum disorder; TD-dep = typically developing adults with current depressive disorders; TD-con = typically developing comparison adults with no history of depression or anxiety.

Because we did not observe significant effects of emotion condition within cohorts, it was not clear at this point if participants were responding to the emotional stimuli as intended by the task. However, in testing our subsequent hypotheses, we found evidence of significant within-group differences in response to specific emotion conditions that was predicted by self-report measures of depression and rumination. This pattern indicates that the NimStim emotional face images were in fact salient stimuli for particular individuals with ASD, in keeping with our hypotheses. This is described next.

### Effects of depressive symptoms and rumination on pupil responsivity over time within ASD

**Fig 4:**
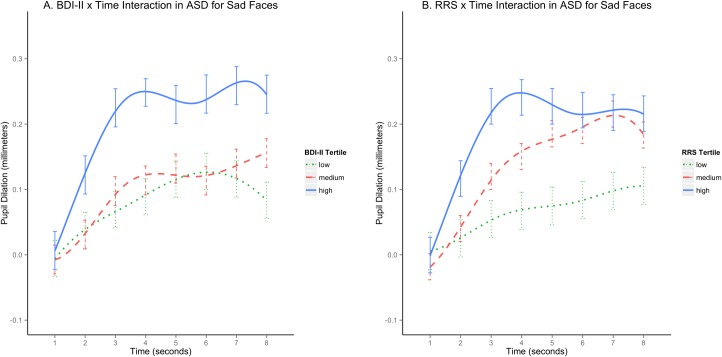
***Relation between pupil dilation to sad stimuli across trial duration and depressive symptoms (A) and rumination (B) within ASD*.** ASD = adults with autism spectrum disorder; BDI-II = Beck Depression Inventory, 2^nd^ edition; RRS = Ruminative Response Scale. Both y-axes represent individual-baseline corrected pupil magnitude in millimeters on a consistent scale (-0.1 to +0.40), with higher scores used here to operationalize greater cognitive-affective responsivity. Both x-axes depict the 8 seconds of trial duration. To interpret the BDI-II X Time and RRS X Time interactions, subgroups were formed by splitting the ASD group into BDI-II tertiles (low: BDI<3, medium: BDI≥3 and <16, high: BDI≥16) and RRS tertiles (low: RRS Total<35, medium: RRS Total ≥35 and <44, high: RRS Total≥44). Standard errors for pupil response at each second along the 8 second interval were estimated by computing least squares means for the estimated pupil curves at the mean BDI-II and RRS value for each tertile (note that LSMEANS within PROC MIXED automatically adjusts for multiple comparisons).

We next assessed the relationship between depressive symptoms and pupil response over time within the ASD cohort, with initial focus on responses to sad stimuli in line with our *a priori* hypotheses. A mixed effects analysis on pupil responses to Sad yielded a significant depression X time interaction (F(7, 112) = 2.25, p = .035), with “time” referring to the 8 seconds of viewing the scrambled mask. To clarify this effect, we plotted (smoothed) pupil responses over time for three subgroups formed by dividing ASD participants at tertiles of their BDI-II distribution (0–33%, 33–67%, 67–100%). As shown in Fig 4A, the high BDI-II subgroup within ASD displayed a sharper acceleration and reached a higher peak than the other two subgroups by the 4 second mark, after which responses were sustained. The low and medium BDI-II subgroups within ASD were largely indistinguishable.

The rumination X time interaction on pupil responses to sad stimuli in ASD also was significant (F(7,105) = 2.11; p = .048). As shown in Fig 4B, the pattern of effects for the high and low tertiles of RRS scores was similar to the respective BDI-II subgroups reported above. However, the medium ruminative subgroup demonstrated a more moderate but consistent increase in pupil magnitude and reached a peak comparable to the high ruminative subgroup at second 7.

**Fig 5:**
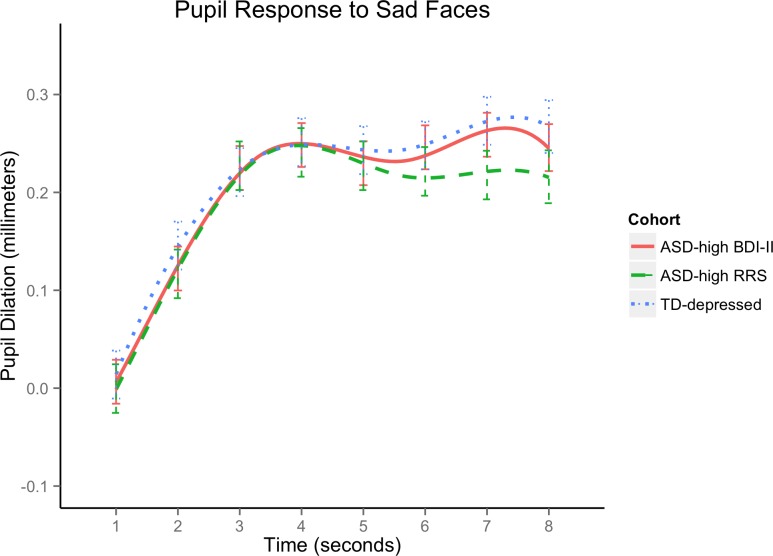
Pupil dilation in response to sad faces in TD-depressed and ASD+elevated depression and/or rumination scores. ASD-high BDI-II = adults with autism spectrum disorder in the high depressive symptoms tertile (BDI-II ≥ 16; BDI-II = Beck Depression Inventory, 2^nd^ edition); ASD-high RRS = adults with autism spectrum disorder in the high rumination tertile (RRS Total ≥ 44; RRS = Ruminative Response Scale); TD-depressed = typically developing adults with current depressive disorders.

We plotted the overlapping subgroups of highest-BDI-II and highest-RRS tertiles alongside the TD-depressed response to Sad stimuli in Fig 5. Standard error bars at each second were adjusted for multiple comparisons and indicate that these three groups marked by elevated depressive symptoms are not significantly different in pupil dilation to sad stimuli over the 8-second trials.

To assess whether these findings were specific to the sad condition, we next conducted exploratory analyses testing BDI-II or RRS interactions with pupil responses to Angry, Happy, and Neutral stimuli and found no significant effects for these other task conditions in ASD. See S1 File for equivalent within-group analyses for typically-developing participants.

### Unique contribution of depressive symptoms and rumination on pupil responsivity to sad stimuli in ASD

To elucidate potential mechanisms linking repetitive thinking and depression in ASD, we assessed the unique and shared contribution of depressive symptoms and rumination on early versus sustained pupil dilation to sad stimuli. Again, because some of these analyses included both variables in the same model, we used the RRS-Brooding subscale here to potentially reduce the common variance with the BDI-II. For early pupil response (seconds 2–3 averaged), both BDI-II scores alone and RRS-Brooding alone significantly predicted pupil response (t(15) = 3.82, p = .002 R^2^ = 0.50; t(15) = 2.17, p = 0.046, R^2^ = .24 respectively). When both predictors were included in the model, the overall R^2^ (= 0.50) was essentially identical to that of the model including only the BDI-II; BDI-II remained significant (t(14) = 2.73, p = 0.016) while RRS-Brooding was not (p>.60). The unique proportion of variance contributed by the BDI-II was estimated at 0.26, the unique proportion to the RRS-Brooding was 0.01, and the remainder (.23) was due to the shared effects of each.

For late pupil response (averaged at seconds 5–8), BDI-II scores alone were significant predictors of pupil response (t(15) = 2.17, p = 0.046, R^2^ = .24) and RRS-Brooding alone showed a trend-level association (t(15) = 1.84, p = 0.09, R^2^ = .18). When both predictors were included, the overall R^2^ in the model (= .24) was again almost identical to the model including BDI-II alone. However, in this model, neither predictor was significant (BDI-II: t(14) = 1.06, p = .31, RRS-Brooding: t(14) = .315, p = .76). Unique proportions of variance were estimated at 0.06 for the BDI-II and 0.005 for RRS-Brooding, with the remainder (.18) due to their shared variance.

## Discussion

People with ASD who had elevated rumination tended to also have elevated depressive symptoms and pupil response patterns to sad stimuli that were comparable to typically developing depressed adults (see Fig 5). These novel findings indicate that individuals with ASD who ruminate exhibit psychophysiological processes that are similar to those of typically developing individuals with depression. Treatments for depression in ASD thus may profit from following models used in other ruminative populations (e.g., typically developing depressed), rather than approaching negative repetitive thinking in ASD as another marker of autism-specific stereotypy.

In line with our first hypothesis, this ASD sample was significantly higher than never-depressed TD-controls and significantly lower than currently depressed TD adults on self-reported rumination and depressive symptoms. However, ASD and TD-depressed did not consistently differ on measures of broader restricted and repetitive behavior, and rumination scores were significantly correlated with these measures within ASD and within TD participants not stratified by depression status (S2 and S5 Tables). This suggests depressive rumination may be similar in presentation and/or function to cognitive perseveration underlying traditional autism-associated repetitive behaviors. Finally, as in other studies [6, 26, 38], rumination and depressive symptoms also were highly correlated both within ASD (r = .80) and non-ASD participants (r = .78).

Per our second hypothesis, TD-depressed participants showed immediate high pupil response to emotional material compared to controls, and tended to sustain response magnitude across the 8 second trials. ASD generally resembled TD-controls in low initial pupil response but approached the TD-depressed group in greater magnitude by trial end. This ASD pupil curve seems unlikely to represent a purely physiological difference in pupil functioning specific to ASD: Greater latency of the pupillary light reflex has been documented in ASD [50] but in child samples (in which pupil response is subject to developmental effects) and on the order of milliseconds that would not affect the current results. An alternative explanation of the observed ASD response could be a pattern of social-emotional processing that is initially disorganized (thus more gradual ramp-up) but ultimately more effortful, as observed in previous ASD literature [25]. However, our subsequent analyses suggest that this “autism-specific” profile of emotional processing results from heterogeneity of mood and repetitive thinking within ASD.

### Specifying the roles of rumination and depressive symptoms in ASD emotion processing

Supporting our third hypothesis, higher levels of depressive symptoms and rumination were each associated with greater pupil dilation over time to sad stimuli within ASD. People in the ASD cohort who ranked in the highest third on depressive symptoms and/or elevated rumination had faster, larger, and sustained pupil response to sad faces compared to their lesser-depressive or -ruminative peers (Fig 4), which markedly resembled the TD-depressed pupil course to Sad (Fig 5). Higher initial and sustained response to dysphoric stimuli apparently represents a similarity in emotion processing across ASD and TD populations that is specific to those with mood disturbances. There was a great deal of overlap in the highest-BDI-II and highest-RRS subgroups; as we cannot differentiate high rumination from high depression-scorers in this ASD sample on either self-report or pupil response, rumination appears to function similarly to general depressive rumination, rather than as a form of repetitive negative thinking unique to ASD. This preliminary evidence supports appealing to the existing rumination/depression literature for treatment recommendations in those with ASD and co-occurring depressed mood.

Across disorders, treatment response likely hinges on a combination of addressing deficits and capitalizing on capabilities. Here we have explored rumination with the goal of informing emotional health interventions in ASD. Common interventions in ruminative populations focus on distraction, reappraisal, and nonjudgmental awareness and tolerance of negative thoughts [1]. In subsequent work we can look at how to modify these or similar “active ingredients” in light of both the strengths and challenges common to the ASD population. Our findings also underscore the need to explore within ASD other mechanisms underlying the development and maintenance of depression that are well-documented in the general literature (e.g., biased attention to negative stimuli, difficulty disengaging from negative stimuli, negative cognitive attributions) to identify other target mechanisms for treatment.

Our fourth hypothesis was partially supported: Depressive symptoms uniquely predicted early pupil response to sad faces, while rumination and depression scores each predicted sustained pupil response in separate models, cancelling the effect of each other within the same model. This joint effect is likely due to the high correlation between these symptoms, but may reflect overall greater sustained pupil dilation for individuals with high levels of both rumination and depressive symptoms (Fig 4). In contrast, participants with ASD in the middle rumination tertile were characterized by notable levels of rumination *without* particularly high depression scores (BDI-II M = 7.5, SD = 7.6; range 0–21). Thus, examining the pupil curve for “medium” level ruminators (red hatched line in Fig 4B) may be more accurate in reflecting the unique effect of rumination on sustained pupil dilation. Strikingly, this subgroup isolates the general “autism processing” pattern noted in previous psychophysiological literature (slower initial but overall heightened response to emotional material, which may represent more effortful neural processing of emotion in ASD)[25]. These findings keep alive the hypotheses that sustained neural engagement with emotional stimuli may bias a process that leads to repetitive thinking in ASD—or conversely, that proclivity for repetitive thinking may lead to sustained neural processing of emotional stimuli. If longer-term ruminative processes follow this latency pattern, thus building more slowly in ASD, we should test the utility of diverting attention (possibly through learned coping strategies) or enhancing predictability of emotional stimuli, in either case to “check” response magnitude early, which may in turn prevent perseveration.

Previous conclusions that the ASD population is initially slow in emotion processing could be subject to latent effects similar to our observed effects in response to sad stimuli: the overall slow-but-sustained pattern linked with ASD may be specifically related to repetitive thinking, whereas a unique pattern of fast-and-sustained cognitive-affective response may be driven by elevated depressive symptoms even within ASD. This reminds us of the value of stratifying adolescent or adult samples on depressive symptoms and related variables to increase validity of findings in broader ASD research. See Herrington et al. [51] for an analogous example in which covarying anxiety enhanced clarity of interpretation in an autism neuroscience study.

Another future direction is to explore how these findings relate to the theory of empathic overarousal in individuals with ASD, which may be mediated by subcortical hyperactivation in response to certain aspects of social stimuli [52, 53]. This theory may be supported by our finding of overall greater average pupil magnitude in the ASD cohort (compared to controls) across emotion condition, e.g., high responsivity to both happy and angry images. The fact that our TD-depressed group displayed similar heightened response across emotion condition, coupled with the fact that the ASD responses varied by internalizing symptoms, may alternatively hint at a form of mood-related “emotion context insensitivity,” as observed previously in samples with major depressive disorder [54]—though this often accompanies an overall blunted effect regardless of emotion condition, rather than a heightened response. Both of these theories warrant further investigation in light of the current findings.

### Limitations

Our small within-cohort sample sizes may limit our power to detect some effects. The depressive symptoms and rumination x time interactions yielded significant effects only for pupil response to *sad* images, which supports our general line of investigation, however other emotion conditions may have reached significances in larger samples. Next, neutral faces could have functioned as a negative stimulus for some participants, in line with previous findings within ASD [55, 56] and other populations [57, 58]. Nevertheless, we were able to note specific effects for sad stimuli even without a reliably neutral comparison condition. We also note that self-report within ASD may be affected by individual impairments in insight, self-awareness, and Theory of Mind. However, in demographically similar samples, independent research teams have found evidence for the importance of self-report of mood over caregiver reports [59]. Our own team has found that the BDI-II in particular makes significant contributions to clinical diagnosis of a mood disorder over other depression scales within young adults with ASD [60]. Self-report was deemed necessary to operationalize internalized mood and thinking patterns in this study, and our results triangulating pupil-by-questionnaire score seem to validate that these self-reports were meaningful in aggregate here. Finally, we did not assess the role of anxiety in relation to rumination, pupil response, and depressive scores, but this represents an important future direction that we hope to explore in a larger dataset with greater power.

## Conclusions

Greater immediate-and-sustained pupil response to dysphoric stimuli appears to be associated with high rumination and depressive symptoms across ASD and TD samples, while the alternative slow-then-sustained response pattern observed here may relate to repetitive thinking within ASD alone. These results suggest that, within ASD, rumination in the context of depression is generally “depression-like,” with similar neural signatures and correlates. We conclude that depression-specific treatments in ASD are likely to profit from following models used in other ruminative populations. This is a valuable starting point, given the prevalence and impairment associated with co-occurring depressed mood in ASD and the lack of evidence-based treatment recommendations. Future work is needed to test the efficacy of “off-the-shelf” or adapted treatments for depression in ASD. We also hope to explore whether our pupil response findings indicate shared phenomenology of current depression versus shared mechanism of *risk* for depression across TD and ASD, and what role if any that residual markers of prior depressive episodes play in either scenario.

In addition to these novel clinical implications, we hope this work contributes to the study of *shared* mechanisms underlying autism and mood disorders. Depression is more common in first-degree relatives of individuals with ASD than in controls, even prior to the birth of a child with ASD [61–63]. To our knowledge, this manuscript represents the only study to directly compare psychophysiological markers of emotion processing across adults with ASD and typically developing currently-depressed adults, versus never-depressed controls. Further, stratifying by self-reported depressive symptoms and rumination suggested clear subtypes within our ASD cohort on pupil-indexed neural processing of emotional stimuli. These preliminary findings reinforce the value of exploring (or controlling for) internalizing and related variables when conducting neurobiological or social-emotional processing research within the heterogeneous autism population.

## Supporting information

S1 File Supporting information referenced in text. (DOCX)

S2 File Original data regarding eye gaze location in exploration of the 400 millisecond presentation of face stimuli. This dataset may be used to recreate heatmaps of face exploration to verify consistency of attention to face stimuli across conditions and across diagnostic cohorts (those heatmaps also available upon request from the Corresponding Author, K.O.G.). Variable “MediaName” references the specific NimStim (Tottenham, 2009) image for each trial.
(XLSX)

S3 File Original demographic, self-report, and pupil data upon which the current Results are based. (CSV)

S1 Fig **Relation between pupil dilation to sad stimuli across trial duration and depressive symptoms (A) and rumination (B) in typically developing depressed adults regardless of depression status.** *Note*. BDI-II = Beck Depression Inventory, 2^nd^ edition; RRS = Ruminative Response Scale; TD = typically developing; TDC = typically developing comparison adults with no history of depression or anxiety; DEP = typically developing adults with current depressive disorders.
Both y-axes represent individual-baseline corrected pupil magnitude in millimeters on a consistent scale (-0.1 to +0.40), with higher scores used here to operationalize greater cognitive-affective responsivity. Both x-axes depict the 8 second mask duration. To interpret the BDI-II X Time and RRS X Time interactions, subgroups were formed by splitting the combined TD group into BDI-II tertiles (Low: BDI-II<2, Medium: > = 2 and <17, High: > = 17) and RRS tertiles (Low: RRS Total<29, Medium: > = 29 and <48, High: > = 48). Standard errors for pupil response at each second along the 8 second interval were estimated by computing least squares means for the estimated pupil curves at the mean BDI-II and RRS value for each tertile (note that LSMEANS within PROC MIXED automatically adjusts for multiple comparisons).
(TIF)

S1 Table Key demographics and self-report descriptives by gender within diagnostic cohorts. *Note*. TD-controls = typically developing control adults with no history of depression or anxiety; ASD = adults with autism spectrum disorder; TD-dep = typically developing adults with current depressive disorders; BDI-II = Beck Depression Inventory, 2^nd^ edition; RRS = Ruminative Response Scale; RBS-R = Repetitive Behavior Scale-Revised overall total score. Individual baseline-corrected raw pupil scores, averaged at seconds 2, 5, and 8 during viewing of the scrambled mask following sad stimuli, are presented here to indicate general pupil response to Sad by gender.
(DOCX)

S2 Table Correlations between demographic and psychometric variables within ASD. *Note*: BDI-II = Beck Depression Inventory, 2^nd^ edition; RRS = Ruminative Response Scale; RRS brooding = Ruminative Response Scale, Brooding subscale; RBS-R Total = Repetitive Behavior Scale-Revised overall total score; SRS-RRB = Social Responsiveness Scale, 2^nd^ edition, Restricted Repetitive Behavior subscale T-score; SRS Total = Social Responsiveness Scale, 2^nd^ edition, overall total score; IS = Interests Scale overall “Intensity” score. Bold type indicates significance at p < .05.
(DOCX)

S3 Table Correlations between demographic and psychometric variables within typically developing depressed adults. *Note*: BDI-II = Beck Depression Inventory, 2^nd^ edition; RRS = Ruminative Response Scale; RRS brooding = Ruminative Response Scale, Brooding subscale; RBS-R Total = Repetitive Behavior Scale-Revised overall total score; SRS-RRB = Social Responsiveness Scale, 2^nd^ edition, Restricted Repetitive Behavior subscale T-score; SRS Total = Social Responsiveness Scale, 2^nd^ edition, overall total score; IS = Interests Scale overall “Intensity” score. Bold type indicates significance at p < .05.
(DOCX)

S4 Table Correlations between demographic and psychometric variables within typically developing never-depressed adults. *Note*: BDI-II = Beck Depression Inventory, 2^nd^ edition; RRS = Ruminative Response Scale; RRS brooding = Ruminative Response Scale, Brooding subscale; RBS-R Total = Repetitive Behavior Scale-Revised overall total score; SRS-RRB = Social Responsiveness Scale, 2^nd^ edition, Restricted Repetitive Behavior subscale T-score; SRS Total = Social Responsiveness Scale, 2^nd^ edition, overall total score; IS = Interests Scale overall “Intensity” score. Bold type indicates significance at p < .05.
(DOCX)

S5 Table Correlations between demographic and psychometric variables within all typically developing participants (depressed and never-depressed combined). *Note*: BDI-II = Beck Depression Inventory, 2^nd^ edition; RRS = Ruminative Response Scale; RRS brooding = Ruminative Response Scale, Brooding subscale; RBS-R Total = Repetitive Behavior Scale-Revised overall total score; SRS-RRB = Social Responsiveness Scale, 2^nd^ edition, Restricted Repetitive Behavior subscale T-score; SRS Total = Social Responsiveness Scale, 2^nd^ edition, overall total score; IS = Interests Scale overall “Intensity” score. Bold type indicates significance at p < .05.
(DOCX)
