# Supplementary material for: Pupil response to social-emotional material is associated with rumination and depressive symptoms in adults with autism spectrum disorder
Source: PLoS One. 2018 Aug 7;13(8):e0200340. doi: 10.1371/journal.pone.0200340 (PMC6080759; doi:10.1371/journal.pone.0200340)
Supplement: S1 File — (DOCX) [file pone.0200340.s001.docx]

**S1 File.**

**Supplementary Materials to “*Pupil response to social-emotional material is associated with rumination and depressive symptoms in adults with autism spectrum disorder”***

*Demographic differences by cohort*

These data are not group-matched across cohorts and differ significantly on several individual characteristics (see main text Table 1, column “Group Differences”). However, the demographic variables that differed significantly by cohort do not appear to exert meaningful influence on our variables of interest either empirically (see S1-S4 Tables below) or by theory. For example, the autism spectrum disorder (ASD) cohort was significantly younger than the typically developing (TD) never-depressed control cohort. However, age has minimal effects on pupil response after childhood [1]. Second, when coupled with the fact that Beck Depression Inventory (BDI-II) [2] and Ruminative Response Scale (RRS) [3] scores tended to be greater in older participants with ASD (which replicates previous findings [4]), this indicates that we would be less likely to detect effects in the ASD group (Type II error). Yet we found the most robust associations between rumination, depression, and pupil scores in the ASD cohort over the others, and thus are continuing to report on these novel data.

Other group differences increase generalizability to these populations under comparison: From base rates of both disorders, we would expect to have more men represented in the ASD group and more women in the depressed cohort [5, 6]. From all indications in the literature, rumination is more prevalent in women than in men (in many samples, this mediates the sex effect in which more women than men become depressed [7]), however, the *mechanism* is not thought to function differently across men and women, such that, when rumination is present in males, it also tends to be associated with depression [7, 8]. In future analyses with larger sample sizes, we will assess for gender effects. Given the absence of power to do so here, we present means and standard deviations on key demographic and exposure variables by gender within each cohort in S1 Table.

Finally, Verbal IQ is often lower in “traditional” ASD samples (versus later-diagnosed, milder samples) than controls, however all participants had VIQs exceeding 80, and Nonverbal IQ was not significantly different across cohorts.

*Additional information on data collection procedures*

Whenever possible, pupil data were collected first to avoid fatigue and bias and to maximize usable data collection. The Wechsler Abbreviated Scale of Intelligence (WASI-II) [9] and untimed Passage Comprehension subtest of the Woodcock Johnson IV, Tests of Achievement (WJ) [10] were used to assess IQ and reading level.

The Autism Diagnostic Observation Schedule (ADOS-2) [11] was administered to all participants in the ASD cohort to confirm diagnosis, as well as to any participants who exceeded clinical cut-points on the Social Responsiveness Scale (SRS-2) [12] or Autism Spectrum Quotient (AQ) [13]. One participant who screened into an ADOS-2 in this way was followed up with an Autism Diagnostic Interview-Revised (ADI-R) [14] and ultimately given a first-time ASD diagnosis. Neurotypical (i.e., non-ASD) status was confirmed in this way for other participants who exceeded ASD screening instrument cut-offs (n=3).

*Pupil task description*

See Figure 1 in main text for visual depiction of this passive-viewing task in which emotionally-salient faces were briefly presented, then masked. A fixation cross first was displayed for 1, 2.5, or 4 seconds (randomly jittered across trials to avoid anticipatory responses and promote continued attention), followed by a single image of a face for 400 milliseconds. Face images were presented in random order across 20 actors (10 women, 10 men) from the NimStim set [15], each performing four expressions: Happy, Sad, Angry, and Neutral. In each of the resulting 80 trials, the face image was followed by a scrambled mask (pixelated image) shown for 8 seconds. The mask was a pixelated image created in Photoshop with same mean luminance as both face slides and fixation cross slides. All slides were shown in grayscale, and all were matched for mean luminance and low-level salient aspects, such as color and contrast, using the Spectral Visual Saliency Toolbox in Matlab [16, 17]. The NimStim faces were selected on the basis of matching standardized ratings of emotional prototypicality from the norming sample; images were cropped to remove hair and non-facial features. The 80 trials were separated into three blocks; participants were given breaks between these blocks and recalibrated on the eye-tracker prior to each block. Prior to the task, participants were told that they would “see many different pictures on the computer. The only instruction is to keep your eyes on the computer screen the whole time until I tell you it’s okay to look away.” After the task, participants completed dimensional valence and arousal ratings of the face images, though these data are not available for all participants due to time constraints.

*Pupil data acquisition & cleaning*

Pupil tasks were displayed on a 24 inch/61cm (diagonal) monitor in a dimly lit, sparsely furnished room with few distractions. Participants were seated 55 cm from the screen. They continued to wear any vision-correcting tools typically needed in their daily lives (glasses, contact lenses). A Tobii X2-60 Hz eye-tracker recorded pupil data, which allowed for non-invasive measurement and relatively free head movement (although participants were asked to remain as still as possible and keep their eyes on the screen at all times unless explicitly told to take a break). Tobii hardware illuminates the pupil via infrared light sources and uses two image sensors to record the reflection patterns. The eye-tracker collected data every 16.67 ms and was (re)calibrated before each block of trials using a five-point calibration screen. All measurements in this paper refer to horizontal pupil diameter in millimeters. For all participants, pupil dilation at all time points within a single trial was corrected for the individual’s baseline pupil measurement, as defined by the average of the last 100 ms of viewing the fixation cross preceding the trial stimulus.

Blinks were identified as samples with estimated pupil diameter meeting any of the following criteria, taken from Siegle et al. [18]: (1) below 1 mm, (2) below the minimum diameter in a subject's waveform + 0.1 mm, (3) below the median diameter minus 4 mm, (4) below two times the interquartile range below the 25th percentile (i.e., the Tukey extreme outlier hinge), or (5) samples with changes in pupil diameter above 0.4 mm in four samples (66 ms). When two blinks were separated by an interval of less than 10 samples, both blinks and the interval between them were defined as part of a single blink. Linear interpolations beginning four samples before and ending nine samples after a blink replaced blinks throughout the dataset (see [18]). Trials comprised of over 50% blinks were excluded from further analysis; participants whose data were excluded for greater than 50% of the 80 possible trials were excluded from pupil analyses. These procedures resulted in the elimination of 2 participants with ASD and 1 TD-control participant for this task.

*Pupil data analysis*

To assess for differences in individual baseline-corrected pupil dilation magnitude by diagnostic cohort, across time (seconds 1 to 8) and emotion category of stimulus (e.g., Happy, Sad, Angry, Neutral face images), we tested for the main and interactive effects of cohort, emotion condition, and second on mean pupil dilation measures using the marginal linear model for correlated response data in SAS PROC MIXED [19, 20]. Restricted maximum likelihood (REML) was used for estimation and general F statistics with Kenward-Roger degrees of freedom were used for tests of fixed effects to minimize biases in estimation. A Kronecker Product (KP) structure [21] on the residual covariance matrix was used to model the within-subject dependency in pupil dilation across seconds and due to emotion condition. An unrestricted covariance structure was used to model the covariance across emotion conditions, and an autoregressive lag 1 structure was used to model the covariances among pupil responses across seconds to model non-independence. To examine specific contrasts, we conducted cohort x second simple effects analyses from seconds 1 to 8 to examine group differences in pupil dilation throughout the 8-second interval. A Bonferroni-adjusted significance value [22] was used to control for multiplicity effects.

*Association between self-report variables across cohorts*

Within ASD (S2 Table below) and within all non-ASD participants combined (typically-developing depressed and never-depressed controls; S5 Table), the RRS and BDI-II were significantly associated with each other, with several subscales that measured repetitive behaviors and intense fixed interests, and with a dimensional measure of autism symptoms (SRS-2 Total). Few significant correlations of interest were noted in the individual typically developing comparison groups that had been stratified a priori on depression status (TD-depressed and TD-controls; S3-S4 Tables).

*Effects of depressive symptoms and rumination on pupil responsivity over time within typically developing adults*

We wished to comment on the relationship between BDI-II and RRS scores and pupil response over time within the typically developing cohorts (TD-depressed and TD-controls) as a comparison for the ASD results presented in the main text. Combining all typically developing participants regardless of depression status more closely approximates the variability of these constructs that we would expect to see in the general population. As we would hypothesize, rumination indeed was more highly associated with pupil dilation in the context of this greater variability in both RRS scores and pupil response, compared to within the TD-depressed or TD-control cohorts alone (similar to the correlational results described above). However, our method of stratifying into depressed and never-depressed cohorts during subject recruitment creates a bi-modal distribution on depressive symptoms when the cohorts are collapsed. This likely also affects the variability of RRS scores, given their high correlation with the BDI-II.  For this reason, combining TD participants creates broader but still artificially restricted distributions of both predictors, and thus these analyses and graphs of their relations to pupil dilation are presented as exploratory only.

In the combined TD sample, BDI-II x Time interaction was significant for Happy (F(7, 203)=3.04, p=.0047) and Sad (F(7, 203)=2.06, p=.0499) conditions. For consistency with the ASD results in the main text (Figure 4), we plotted pupil responses to Sad stimuli over time for three subgroups formed by dividing all TD participants at tertiles of their BDI-II distribution (0-33%, 33-67%, 67-100%). As shown in S1.A Fig, individuals in the high tertile of BDI-II scores had the highest averaged pupil dilation across time. Contrary to expectations, participants in the low BDI-II subgroup (all 9 of whom had scores of 0 or 1 on the BDI-II) tended to have significantly higher pupil dilation to Sad stimuli at most time points compared to those in the medium subgroup (10 people with BDI-II scores between 2 and 16); our current sample size is not sufficient to fully explore possible moderators of this effect.

RRS x Time interaction was significant for Angry (F(7, 203)=2.10, p=.0450), Happy (F(7, 203)=3.93, p=.0005), and Sad (F(7, 203)=3.26, p=.0026) conditions. When plotting tertiles against pupil response to Sad (S1.B Fig), the high RRS subgroup displays a sharper acceleration and reaches a higher peak than the other two subgroups by the 4 second mark, after which responses are sustained. The medium subgroup has an initial pupil response more similar to the high RRS group than the low RRS group, after which the pupil courses of the low and medium RRS subgroups are largely indistinguishable.

*References for Supplementary Materials*

1. Daluwatte C, Miles JH, Christ SE, Beversdorf DQ, Lofgreen A, Berliner N, et al. Age-dependent pupillary light reflex parameters in children. In: 2012 Annual International Conference of the IEEE Engineering in Medicine and Biology Society. 2012. p. 3776–9.

2. Beck AT, Steer RA, Brown GK. Beck Depression Inventory-II (BDI-II). San Antonio TX Psychol Corp. 1996.

3. Nolen-Hoeksema S, Morrow J. A prospective study of depression and posttraumatic stress symptoms after a natural disaster: the 1989 Loma Prieta Earthquake. J Pers Soc Psychol. 1991;61:115.

4. Gotham K, Bishop SL, Brunwasser S, Lord C. Rumination and perceived impairment associated with depressive symptoms in a verbal adolescent–adult ASD sample. Autism Res. 2014;7:381–91.

5. Fombonne E. Epidemiological surveys of autism and other pervasive developmental disorders: An update. J Autism Dev Disord. 2003;33:365–82.

6. Kessler RC. Epidemiology of women and depression. J Affect Disord. 2003;74:5–13.

7. Nolen-Hoeksema S, Wisco BE, Lyubomirsky S. Rethinking rumination. Perspect Psychol Sci. 2008;3:400–24.

8. Mezulis A, Simonson J, McCauley E, Stoep AV. The association between temperament and depressive symptoms in adolescence: Brooding and reflection as potential mediators. Cogn Emot. 2011;25:1460–70.

9. Wechsler D. Wechsler Abbreviated Scale of Intelligence -- Second Edition (WASI-II). San Antonio, TX: NCS Pearson; 2011.

10. Schrank FA, Mather N, McGrew KS. Woodcock-Johnson IV Tests of Achievement. Roll Meadows IL Riverside. 2014.

11. Lord C, Rutter M, DiLavore PC, Risi S, Gotham K, Bishop S. Autism Diagnostic Observation Schedule: ADOS-2. Western Psychological Services. 2012.

12. Constantino JN, Gruber CP. Social Responsiveness Scale (SRS-2). Western Psychological Services Los Angeles, CA; 2007.

13. Baron-Cohen S, Wheelwright S, Skinner R, Martin J, Clubley E. The Autism-Spectrum Quotient (AQ): Evidence from Asperger syndrome/high-functioning autism, males and females, scientists and mathematicians. J Autism Dev Disord. 2001;31:5–17.

14. Lord C, Rutter M, Le Couteur A. Autism Diagnostic Interview-Revised: A revised version of a diagnostic interview for caregivers of individuals with possible pervasive developmental disorders. J Autism Dev Disord. 1994;24:659–685.

15. Tottenham N, Tanaka JW, Leon AC, McCarry T, Nurse M, Hare TA, et al. The NimStim set of facial expressions: Judgments from untrained research participants. Psychiatry Res. 2009;168:242–9.

16. Schauerte B, Stiefelhagen R. Quaternion-based spectral saliency detection for eye fixation prediction. In: Computer Vision – ECCV 2012. Springer, Berlin, Heidelberg; 2012. p. 116–29.

17. Guide MU. The Mathworks. Inc Natick MA. 1998;5:333.

18. Siegle GJ, Ichikawa N, Steinhauer S. Blink before and after you think: Blinks occur prior to and following cognitive load indexed by pupillary responses. Psychophysiology. 2008;45:679–87.

19. Littell RC, Stroup WW, Milliken GA, Wolfinger RD, Schabenberger O. SAS for mixed models. SAS institute; 2006.

20. Verbeke G, Molenberghs G. Linear mixed nodels for longitudinal data. Springer Science & Business Media; 2009.

21. Galecki AT. General class of covariance structures for two or more repeated factors in longitudinal data analysis. Commun Stat - Theory Methods. 1994;23:3105–19.

22. Westfall PH, Tobias RD, Wolfinger RD. Multiple comparisons and multiple tests using SAS. SAS Institute; 2011.
