# Supplementary figures and images for: Pupil response to social-emotional material is associated with rumination and depressive symptoms in adults with autism spectrum disorder
Source: PLoS One. 2018 Aug 7;13(8):e0200340. doi: 10.1371/journal.pone.0200340 (PMC6080759; doi:10.1371/journal.pone.0200340)

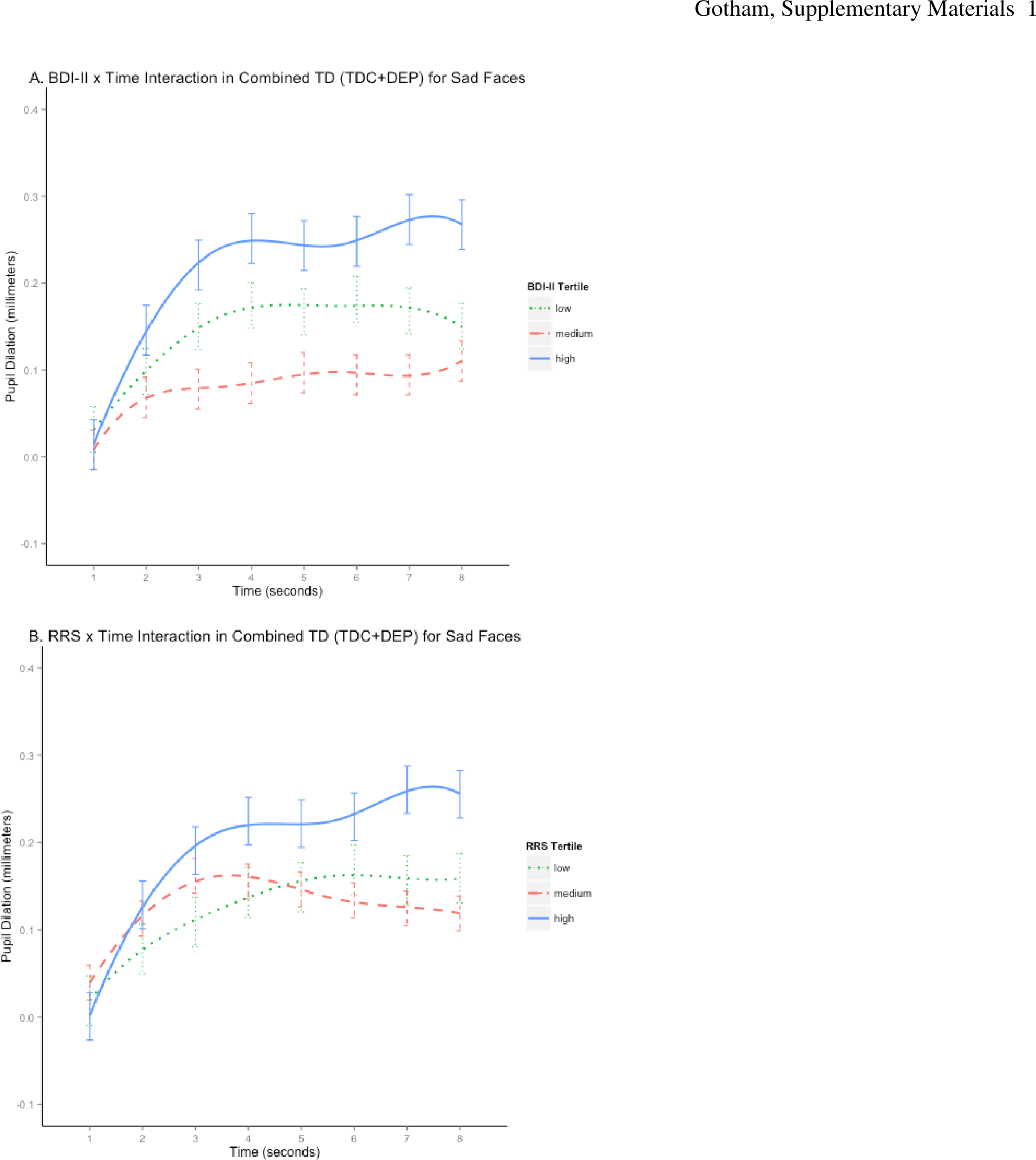

Supplement: S1 Fig — Relation between pupil dilation to sad stimuli across trial duration and depressive symptoms (A) and rumination (B) in typically developing depressed adults regardless of depression status. Note. BDI-II = Beck Depression Inventory, 2nd edition; RRS = Ruminative Response Scale; TD = typically developing; TDC = typically developing comparison adults with no history of depression or anxiety; DEP = typically developing adults with current depressive disorders. Both y-axes represent individual-baseline corrected pupil magnitude in millimeters on a consistent scale (-0.1 to +0.40), with higher scores used here to operationalize greater cognitive-affective responsivity. Both x-axes depict the 8 second mask duration. To interpret the BDI-II X Time and RRS X Time interactions, subgroups were formed by splitting the combined TD group into BDI-II tertiles (Low: BDI-II<2, Medium: > = 2 and <17, High: > = 17) and RRS tertiles (Low: RRS Total<29, Medium: > = 29 and <48, High: > = 48). Standard errors for pupil response at each second along the 8 second interval were estimated by computing least squares means for the estimated pupil curves at the mean BDI-II and RRS value for each tertile (note that LSMEANS within PROC MIXED automatically adjusts for multiple comparisons). (TIF) [file pone.0200340.s004.tif]
