# Supplementary material for: Pupil response to social-emotional material is associated with rumination and depressive symptoms in adults with autism spectrum disorder
Source: PLoS One. 2018 Aug 7;13(8):e0200340. doi: 10.1371/journal.pone.0200340 (PMC6080759; doi:10.1371/journal.pone.0200340)
Supplement: S1 Table — Note. TD-controls = typically developing control adults with no history of depression or anxiety; ASD = adults with autism spectrum disorder; TD-dep = typically developing adults with current depressive disorders; BDI-II = Beck Depression Inventory, 2nd edition; RRS = Ruminative Response Scale; RBS-R = Repetitive Behavior Scale-Revised overall total score. Individual baseline-corrected raw pupil scores, averaged at seconds 2, 5, and 8 during viewing of the scrambled mask following sad stimuli, are presented here to indicate general pupil response to Sad by gender. (DOCX) [file pone.0200340.s005.docx]

*S1 Table. Key demographics and self-report descriptives by gender within diagnostic cohorts*

| **Mean(SD)**  **Range** | **TD-controls** | | **ASD** | | **TD-dep** | |
| --- | --- | --- | --- | --- | --- | --- |
|  | Female | Male | Female | Male | Female | Male |
| N | 11 | 8 | 4 | 17 | 7 | 5 |
| Age  in Years | 28.0(4.5)  22—35 | 24.8(4.0)  21—32 | 24.0(6.1)  20—33 | 22.1(4.4)  18—33 | 23.1(2.6)  19—26 | 24.8(4.4)  22—32 |
| Verbal  IQ | 117.8(14.7)  100—147 | 109.6(13.0)  90—130 | 108.3(9.7)  98—117 | 99.3(10.7)  81—120 | 112.6(10.8)  98—130 | 118.0(4.0)  112—123 |
| Nonverbal IQ | 108.2(12.9)  79—125 | 107.5(18.7)  79—136 | 109.8(6.9)  101—117 | 105.9(16.6)  81—120 | 110.0(11.6)  89—126 | 112.6(7.2)  106—123 |
|  |  |  |  |  |  |  |
| BDI-II | 2.5(2.2)  0—8 | 1.6(2.0)  0—4 | 11.3(12.5)  0—24 | 11.3(9.5)  0—29 | 24.1(6.9)  17—33 | 24.2(6.4)  17—34 |
| RRS | 31.3(8.8)  23—53 | 30.6(7.4)  23—44 | 45.0(20.6)  28—74 | 41.2(11.4)  23—61 | 54.9(5.7)  49—65 | 49.4(7.4)  39—59 |
| RBS-R | 2.4(2.7)  0—8 | 5.7(7.2)  0—15 | 14.8(10.9)  0—24 | 24.5(25.4)  1—79 | 14.3(19.9)  0—56 | 10.0(8.0)  4—23 |
| Pupil to Sad, Second 2 | 0.08(0.09)  -0.1—0.2 | 0.09(0.07)  -0.01—0.2 | 0.06(0.06)  -0.002—0.1 | 0.07(0.09)  -0.1—0.2 | 0.1(0.1)  -0.1—0.3 | 0.2(0.1)  0.02—0.4 |
| Pupil to Sad, Second 5 | 0.12(0.11)  -0.06—0.3 | 0.15(0.10)  0.001—0.3 | 0.2(0.1)  0.1—0.3 | 0.2(0.1)  -0.02—0.5 | 0.3(0.1)  0.1—0.5 | 0.2(0.1)  0.1—0.4 |
| Pupil to Sad, Second 8 | 0.13(0.07)  0.04—0.3 | 0.13(0.11)  0.004—0.3 | 0.2(0.2)  0.03—0.4 | 0.2(0.1)  -0.04—0.4 | 0.3(0.1)  0.1—0.4 | 0.2(0.2)  -0.04—0.4 |

*Note.* TD-controls=typically developing control adults with no history of depression or anxiety; ASD=adults with autism spectrum disorder; TD-dep=typically developing adults with current depressive disorders; BDI-II=Beck Depression Inventory, 2^nd^ edition; RRS=Ruminative Response Scale; RBS-R=Repetitive Behavior Scale-Revised overall total score. Individual baseline-corrected raw pupil scores, averaged at seconds 2, 5, and 8 during viewing of the scrambled mask following sad stimuli, are presented here to indicate general pupil response to Sad by gender.
