# Supplementary material for: Pupil response to social-emotional material is associated with rumination and depressive symptoms in adults with autism spectrum disorder
Source: PLoS One. 2018 Aug 7;13(8):e0200340. doi: 10.1371/journal.pone.0200340 (PMC6080759; doi:10.1371/journal.pone.0200340)
Supplement: S2 Table — Note: BDI-II = Beck Depression Inventory, 2nd edition; RRS = Ruminative Response Scale; RRS brooding = Ruminative Response Scale, Brooding subscale; RBS-R Total = Repetitive Behavior Scale-Revised overall total score; SRS-RRB = Social Responsiveness Scale, 2nd edition, Restricted Repetitive Behavior subscale T-score; SRS Total = Social Responsiveness Scale, 2nd edition, overall total score; IS = Interests Scale overall “Intensity” score. Bold type indicates significance at p < .05. (DOCX) [file pone.0200340.s006.docx]

*S2 Table. Correlations between demographic and psychometric variables within ASD*

| Pearson r | Age | Verbal IQ | Nonverbal | BDI-II | RRS | RRS | RBS-R | SRS-RRB | SRS Total | IS |
| --- | --- | --- | --- | --- | --- | --- | --- | --- | --- | --- |
| p-value |  |  | IQ |  | Total | Brooding |  |  |  | Intensity |
| n |  |  |  |  |  |  |  |  |  |  |
| Age |  |  |  |  |  |  |  |  |  |  |
|  | - |  |  |  |  |  |  |  |  |  |
|  |  |  |  |  |  |  |  |  |  |  |
| Verbal IQ | **0.540** |  |  |  |  |  |  |  |  |  |
|  | 0.012 | - |  |  |  |  |  |  |  |  |
|  | 21 |  |  |  |  |  |  |  |  |  |
| Nonverbal IQ | 0.378 | **0.522** |  |  |  |  |  |  |  |  |
|  | 0.091 | 0.015 | - |  |  |  |  |  |  |  |
|  | 21 | 21 |  |  |  |  |  |  |  |  |
| BDI-II | **0.464** | -0.105 | -0.156 |  |  |  |  |  |  |  |
|  | 0.039 | 0.660 | 0.513 | - |  |  |  |  |  |  |
|  | 20 | 20 | 20 |  |  |  |  |  |  |  |
| RRS Total | **0.496** | -0.023 | -0.277 | **0.801** |  |  |  |  |  |  |
|  | 0.026 | 0.923 | 0.237 | 0.000 | - |  |  |  |  |  |
|  | 20 | 20 | 20 | 20 |  |  |  |  |  |  |
| RRS Brooding | 0.345 | -0.222 | -0.311 | **0.795** | **0.899** |  |  |  |  |  |
|  | 0.137 | 0.347 | 0.182 | 0.000 | 0.000 | - |  |  |  |  |
|  | 20 | 20 | 20 | 20 | 20 |  |  |  |  |  |
| RBS-R | 0.012 | -0.248 | **-0.620** | **0.650** | **0.596** | **0.651** |  |  |  |  |
|  | 0.963 | 0.320 | 0.006 | 0.004 | 0.009 | 0.003 | - |  |  |  |
|  | 18 | 18 | 18 | 18 | 18 | 18 |  |  |  |  |
| SRS-RRB | **0.602** | 0.190 | -0.100 | **0.530** | **0.658** | **0.591** | **0.505** |  |  |  |
|  | 0.005 | 0.421 | 0.676 | 0.016 | 0.002 | 0.006 | 0.032 | - |  |  |
|  | 20 | 20 | 20 | 20 | 20 | 20 | 18 |  |  |  |
| SRS Total | **0.634** | 0.171 | -0.067 | **0.689** | **0.675** | **0.650** | **0.592** | **0.877** |  |  |
|  | 0.003 | 0.472 | 0.779 | 0.001 | 0.001 | 0.002 | 0.010 | 0.000 | - |  |
|  | 20 | 20 | 20 | 20 | 20 | 20 | 18 | 20 |  |  |
| IS intensity | 0.137 | 0.223 | -0.266 | **0.649** | **0.655** | **0.573** | **0.613** | 0.361 | 0.459 |  |
|  | 0.586 | 0.374 | 0.286 | 0.004 | 0.003 | 0.013 | 0.009 | 0.141 | 0.055 | - |
|  | 18 | 18 | 18 | 18 | 18 | 18 | 17 | 18 | 18 |  |

*Note*: BDI-II=Beck Depression Inventory, 2^nd^ edition; RRS=Ruminative Response Scale; RRS brooding=Ruminative Response Scale, Brooding subscale; RBS-R Total=Repetitive Behavior Scale-Revised overall total score; SRS-RRB=Social Responsiveness Scale, 2^nd^ edition, Restricted Repetitive Behavior subscale T-score; SRS Total=Social Responsiveness Scale, 2^nd^ edition, overall total score; IS=Interests Scale overall “Intensity” score. Bold type indicates significance at p<.05.
