# Supplementary material for: Pupil response to social-emotional material is associated with rumination and depressive symptoms in adults with autism spectrum disorder
Source: PLoS One. 2018 Aug 7;13(8):e0200340. doi: 10.1371/journal.pone.0200340 (PMC6080759; doi:10.1371/journal.pone.0200340)
Supplement: S3 Table — Note: BDI-II = Beck Depression Inventory, 2nd edition; RRS = Ruminative Response Scale; RRS brooding = Ruminative Response Scale, Brooding subscale; RBS-R Total = Repetitive Behavior Scale-Revised overall total score; SRS-RRB = Social Responsiveness Scale, 2nd edition, Restricted Repetitive Behavior subscale T-score; SRS Total = Social Responsiveness Scale, 2nd edition, overall total score; IS = Interests Scale overall “Intensity” score. Bold type indicates significance at p < .05. (DOCX) [file pone.0200340.s007.docx]

*S3 Table. Correlations between demographic and psychometric variables within typically developing depressed adults*

| Pearson r | Age | Verbal IQ | Nonverbal | BDI-II | RRS | RRS | RBS-R | SRS-RRB | SRS Total | IS |
| --- | --- | --- | --- | --- | --- | --- | --- | --- | --- | --- |
| p-value |  |  | IQ |  | Total | Brooding |  |  |  | Intensity |
| n |  |  |  |  |  |  |  |  |  |  |
| Age |  |  |  |  |  |  |  |  |  |  |
|  | - |  |  |  |  |  |  |  |  |  |
|  |  |  |  |  |  |  |  |  |  |  |
| Verbal IQ | 0.130 |  |  |  |  |  |  |  |  |  |
|  | 0.671 | - |  |  |  |  |  |  |  |  |
|  | 13 |  |  |  |  |  |  |  |  |  |
| Nonverbal IQ | -0.316 | 0.424 |  |  |  |  |  |  |  |  |
|  | 0.293 | 0.149 | - |  |  |  |  |  |  |  |
|  | 13 | 13 |  |  |  |  |  |  |  |  |
| BDI-II | -0.092 | 0.060 | 0.174 |  |  |  |  |  |  |  |
|  | 0.765 | 0.846 | 0.570 | - |  |  |  |  |  |  |
|  | 13 | 13 | 13 |  |  |  |  |  |  |  |
| RRS Total | -0.275 | -0.068 | 0.392 | 0.214 |  |  |  |  |  |  |
|  | 0.364 | 0.826 | 0.185 | 0.483 | - |  |  |  |  |  |
|  | 13 | 13 | 13 | 13 |  |  |  |  |  |  |
| RRS Brooding | -0.430 | -0.134 | 0.258 | -0.128 | **0.686** |  |  |  |  |  |
|  | 0.142 | 0.661 | 0.395 | 0.677 | 0.010 | - |  |  |  |  |
|  | 13 | 13 | 13 | 13 | 13 |  |  |  |  |  |
| RBS-R | -0.193 | **-0.645** | -0.531 | 0.109 | -0.132 | 0.081 |  |  |  |  |
|  | 0.528 | 0.017 | 0.062 | 0.724 | 0.666 | 0.792 | - |  |  |  |
|  | 13 | 13 | 13 | 13 | 13 | 13 |  |  |  |  |
| SRS-RRB | 0.084 | -0.087 | 0.070 | 0.550 | -0.117 | -0.121 | 0.379 |  |  |  |
|  | 0.784 | 0.778 | 0.821 | 0.052 | 0.703 | 0.695 | 0.202 | - |  |  |
|  | 13 | 13 | 13 | 13 | 13 | 13 | 13 |  |  |  |
| SRS Total | 0.132 | -0.017 | 0.091 | 0.389 | -0.229 | -0.247 | 0.312 | **0.906** |  |  |
|  | 0.666 | 0.956 | 0.767 | 0.189 | 0.451 | 0.416 | 0.299 | 0.000 | - |  |
|  | 13 | 13 | 13 | 13 | 13 | 13 | 13 | 13 |  |  |
| IS Intensity | -0.173 | -0.266 | -0.096 | 0.030 | -0.121 | 0.077 | **0.620** | 0.550 | **0.703** |  |
|  | 0.572 | 0.380 | 0.754 | 0.923 | 0.694 | 0.803 | 0.024 | 0.052 | 0.007 | - |
|  | 13 | 13 | 13 | 13 | 13 | 13 | 13 | 13 | 13 |  |

*Note*: BDI-II=Beck Depression Inventory, 2^nd^ edition; RRS=Ruminative Response Scale; RRS brooding=Ruminative Response Scale, Brooding subscale; RBS-R Total=Repetitive Behavior Scale-Revised overall total score; SRS-RRB=Social Responsiveness Scale, 2^nd^ edition, Restricted Repetitive Behavior subscale T-score; SRS Total= Social Responsiveness Scale, 2^nd^ edition, overall total score; IS=Interests Scale overall “Intensity” score. Bold type indicates significance at p<.05.

*S4 Table. Correlations between demographic and psychometric variables within typically developing never-depressed adults*

| Pearson r | Age | Verbal IQ | Nonverbal | BDI-II | RRS | RRS | RBS-R | SRS-RRB | SRS Total | IS |
| --- | --- | --- | --- | --- | --- | --- | --- | --- | --- | --- |
| p-value |  |  | IQ |  | Total | Brooding |  |  |  | Intensity |
| n |  |  |  |  |  |  |  |  |  |  |
| Age |  |  |  |  |  |  |  |  |  |  |
|  | - |  |  |  |  |  |  |  |  |  |
|  |  |  |  |  |  |  |  |  |  |  |
| Verbal IQ | -0.185 |  |  |  |  |  |  |  |  |  |
|  | 0.448 | - |  |  |  |  |  |  |  |  |
|  | 19 |  |  |  |  |  |  |  |  |  |
| Nonverbal IQ | 0.189 | 0.434 |  |  |  |  |  |  |  |  |
|  | 0.438 | 0.063 | - |  |  |  |  |  |  |  |
|  | 19 | 19 |  |  |  |  |  |  |  |  |
| BDI-II | 0.295 | -0.232 | -0.028 |  |  |  |  |  |  |  |
|  | 0.221 | 0.339 | 0.910 | - |  |  |  |  |  |  |
|  | 19 | 19 | 19 |  |  |  |  |  |  |  |
| RRS Total | -0.096 | 0.021 | -0.213 | 0.147 |  |  |  |  |  |  |
|  | 0.697 | 0.930 | 0.381 | 0.547 | - |  |  |  |  |  |
|  | 19 | 19 | 19 | 19 |  |  |  |  |  |  |
| RRS Brooding | -0.022 | -0.307 | **-0.522** | **0.470** | **0.579** |  |  |  |  |  |
|  | 0.930 | 0.202 | 0.022 | 0.042 | 0.009 | - |  |  |  |  |
|  | 19 | 19 | 19 | 19 | 19 |  |  |  |  |  |
| RBS-R | 0.063 | **-0.525** | -0.110 | 0.291 | -0.272 | -0.144 |  |  |  |  |
|  | 0.805 | 0.025 | 0.664 | 0.242 | 0.274 | 0.568 | - |  |  |  |
|  | 18 | 18 | 18 | 18 | 18 | 18 |  |  |  |  |
| SRS-RRB | -0.143 | -0.064 | 0.063 | **0.712** | 0.176 | 0.354 | 0.166 |  |  |  |
|  | 0.559 | 0.794 | 0.798 | 0.001 | 0.471 | 0.137 | 0.511 | - |  |  |
|  | 19 | 19 | 19 | 19 | 19 | 19 | 18 |  |  |  |
| SRS Total | -0.295 | -0.201 | 0.212 | **0.489** | 0.022 | 0.101 | 0.440 | **0.776** |  |  |
|  | 0.220 | 0.410 | 0.385 | 0.033 | 0.930 | 0.681 | 0.067 | 0.000 | - |  |
|  | 19 | 19 | 19 | 19 | 19 | 19 | 18 | 19 |  |  |
| IS Intensity | -0.361 | -0.289 | -0.073 | 0.223 | 0.057 | 0.341 | 0.333 | 0.336 | **0.603** |  |
|  | 0.142 | 0.244 | 0.775 | 0.363 | 0.823 | 0.166 | 0.178 | 0.173 | 0.008 | - |
|  | 18 | 18 | 18 | 18 | 18 | 18 | 18 | 18 | 18 |  |

*Note*: BDI-II=Beck Depression Inventory, 2^nd^ edition; RRS=Ruminative Response Scale; RRS brooding=Ruminative Response Scale, Brooding subscale; RBS-R Total=Repetitive Behavior Scale-Revised overall total score; SRS-RRB=Social Responsiveness Scale, 2^nd^ edition, Restricted Repetitive Behavior subscale T-score; SRS Total= Social Responsiveness Scale, 2^nd^ edition, overall total score; IS=Interests Scale overall “Intensity” score. Bold type indicates significance at p<.05.

*S5 Table. Correlations between demographic and psychometric variables within all typically developing participants (depressed and never-depressed combined)*

| Pearson r | Age | Verbal IQ | Nonverbal | BDI-II | RRS | RRS | RBS-R | SRS-RRB | SRS Total | IS |
| --- | --- | --- | --- | --- | --- | --- | --- | --- | --- | --- |
| p-value |  |  | IQ |  | Total | Brooding |  |  |  | Intensity |
| n |  |  |  |  |  |  |  |  |  |  |
| Age |  |  |  |  |  |  |  |  |  |  |
|  | - |  |  |  |  |  |  |  |  |  |
|  |  |  |  |  |  |  |  |  |  |  |
| Verbal IQ | **0.311** |  |  |  |  |  |  |  |  |  |
|  | 0.018 | - |  |  |  |  |  |  |  |  |
|  | 57 |  |  |  |  |  |  |  |  |  |
| Nonverbal IQ | 0.182 | **0.392** |  |  |  |  |  |  |  |  |
|  | 0.176 | 0.003 | - |  |  |  |  |  |  |  |
|  | 57 | 57 |  |  |  |  |  |  |  |  |
| BDI-II | -0.026 | -0.059 | 0.007 |  |  |  |  |  |  |  |
|  | 0.847 | 0.664 | 0.958 | - |  |  |  |  |  |  |
|  | 56 | 56 | 56 |  |  |  |  |  |  |  |
| RRS Total | -0.003 | -0.034 | -0.091 | **0.780** |  |  |  |  |  |  |
|  | 0.981 | 0.802 | 0.504 | 0.000 | - |  |  |  |  |  |
|  | 56 | 56 | 56 | 56 |  |  |  |  |  |  |
| RRS Brooding | -0.023 | -0.238 | -0.208 | **0.697** | **0.844** |  |  |  |  |  |
|  | 0.865 | 0.077 | 0.123 | 0.000 | 0.000 | - |  |  |  |  |
|  | 56 | 56 | 56 | 56 | 56 |  |  |  |  |  |
| RBS-R | -0.207 | **-0.476** | **-0.468** | **0.437** | **0.364** | **0.508** |  |  |  |  |
|  | 0.137 | 0.000 | 0.000 | 0.001 | 0.007 | 0.000 | - |  |  |  |
|  | 53 | 53 | 53 | 53 | 53 | 53 |  |  |  |  |
| SRS-RRB | -0.122 | **-0.317** | -0.057 | **0.471** | **0.418** | **0.467** | **0.617** |  |  |  |
|  | 0.368 | 0.017 | 0.675 | 0.000 | 0.001 | 0.000 | 0.000 | - |  |  |
|  | 56 | 56 | 56 | 56 | 56 | 56 | 53 |  |  |  |
| SRS Total | -0.133 | **-0.335** | -0.023 | **0.522** | **0.425** | **0.486** | **0.648** | **0.947** |  |  |
|  | 0.329 | 0.012 | 0.867 | 0.000 | 0.001 | 0.000 | 0.000 | 0.000 | - |  |
|  | 56 | 56 | 56 | 56 | 56 | 56 | 53 | 56 |  |  |
| IS Intensity | -0.256 | **-0.303** | -0.198 | **0.458** | **0.418** | **0.500** | **0.682** | **0.624** | **0.694** |  |
|  | 0.064 | 0.027 | 0.156 | 0.001 | 0.002 | 0.000 | 0.000 | 0.000 | 0.000 | - |
|  | 53 | 53 | 53 | 53 | 53 | 53 | 52 | 53 | 53 |  |

*Note*: BDI-II=Beck Depression Inventory, 2^nd^ edition; RRS=Ruminative Response Scale; RRS brooding=Ruminative Response Scale, Brooding subscale; RBS-R Total=Repetitive Behavior Scale-Revised overall total score; SRS-RRB=Social Responsiveness Scale, 2^nd^ edition, Restricted Repetitive Behavior subscale T-score; SRS Total= Social Responsiveness Scale, 2^nd^ edition, overall total score; IS=Interests Scale overall “Intensity” score. Bold type indicates significance at p<.05.
