# Supplementary material for: Pupil response to social-emotional material is associated with rumination and depressive symptoms in adults with autism spectrum disorder
Source: PLoS One. 2018 Aug 7;13(8):e0200340. doi: 10.1371/journal.pone.0200340 (PMC6080759; doi:10.1371/journal.pone.0200340)
Supplement: S4 Table — Note: BDI-II = Beck Depression Inventory, 2nd edition; RRS = Ruminative Response Scale; RRS brooding = Ruminative Response Scale, Brooding subscale; RBS-R Total = Repetitive Behavior Scale-Revised overall total score; SRS-RRB = Social Responsiveness Scale, 2nd edition, Restricted Repetitive Behavior subscale T-score; SRS Total = Social Responsiveness Scale, 2nd edition, overall total score; IS = Interests Scale overall “Intensity” score. Bold type indicates significance at p < .05. (DOCX) [file pone.0200340.s008.docx]

*S4 Table. Correlations between demographic and psychometric variables within typically developing never-depressed adults*

| Pearson r | Age | Verbal IQ | Nonverbal | BDI-II | RRS | RRS | RBS-R | SRS-RRB | SRS Total | IS |
| --- | --- | --- | --- | --- | --- | --- | --- | --- | --- | --- |
| p-value |  |  | IQ |  | Total | Brooding |  |  |  | Intensity |
| n |  |  |  |  |  |  |  |  |  |  |
| Age |  |  |  |  |  |  |  |  |  |  |
|  | - |  |  |  |  |  |  |  |  |  |
|  |  |  |  |  |  |  |  |  |  |  |
| Verbal IQ | -0.185 |  |  |  |  |  |  |  |  |  |
|  | 0.448 | - |  |  |  |  |  |  |  |  |
|  | 19 |  |  |  |  |  |  |  |  |  |
| Nonverbal IQ | 0.189 | 0.434 |  |  |  |  |  |  |  |  |
|  | 0.438 | 0.063 | - |  |  |  |  |  |  |  |
|  | 19 | 19 |  |  |  |  |  |  |  |  |
| BDI-II | 0.295 | -0.232 | -0.028 |  |  |  |  |  |  |  |
|  | 0.221 | 0.339 | 0.910 | - |  |  |  |  |  |  |
|  | 19 | 19 | 19 |  |  |  |  |  |  |  |
| RRS Total | -0.096 | 0.021 | -0.213 | 0.147 |  |  |  |  |  |  |
|  | 0.697 | 0.930 | 0.381 | 0.547 | - |  |  |  |  |  |
|  | 19 | 19 | 19 | 19 |  |  |  |  |  |  |
| RRS Brooding | -0.022 | -0.307 | **-0.522** | **0.470** | **0.579** |  |  |  |  |  |
|  | 0.930 | 0.202 | 0.022 | 0.042 | 0.009 | - |  |  |  |  |
|  | 19 | 19 | 19 | 19 | 19 |  |  |  |  |  |
| RBS-R | 0.063 | **-0.525** | -0.110 | 0.291 | -0.272 | -0.144 |  |  |  |  |
|  | 0.805 | 0.025 | 0.664 | 0.242 | 0.274 | 0.568 | - |  |  |  |
|  | 18 | 18 | 18 | 18 | 18 | 18 |  |  |  |  |
| SRS-RRB | -0.143 | -0.064 | 0.063 | **0.712** | 0.176 | 0.354 | 0.166 |  |  |  |
|  | 0.559 | 0.794 | 0.798 | 0.001 | 0.471 | 0.137 | 0.511 | - |  |  |
|  | 19 | 19 | 19 | 19 | 19 | 19 | 18 |  |  |  |
| SRS Total | -0.295 | -0.201 | 0.212 | **0.489** | 0.022 | 0.101 | 0.440 | **0.776** |  |  |
|  | 0.220 | 0.410 | 0.385 | 0.033 | 0.930 | 0.681 | 0.067 | 0.000 | - |  |
|  | 19 | 19 | 19 | 19 | 19 | 19 | 18 | 19 |  |  |
| IS Intensity | -0.361 | -0.289 | -0.073 | 0.223 | 0.057 | 0.341 | 0.333 | 0.336 | **0.603** |  |
|  | 0.142 | 0.244 | 0.775 | 0.363 | 0.823 | 0.166 | 0.178 | 0.173 | 0.008 | - |
|  | 18 | 18 | 18 | 18 | 18 | 18 | 18 | 18 | 18 |  |

*Note*: BDI-II=Beck Depression Inventory, 2^nd^ edition; RRS=Ruminative Response Scale; RRS brooding=Ruminative Response Scale, Brooding subscale; RBS-R Total=Repetitive Behavior Scale-Revised overall total score; SRS-RRB=Social Responsiveness Scale, 2^nd^ edition, Restricted Repetitive Behavior subscale T-score; SRS Total= Social Responsiveness Scale, 2^nd^ edition, overall total score; IS=Interests Scale overall “Intensity” score. Bold type indicates significance at p<.05.
