# Supplementary material for: Pupil response to social-emotional material is associated with rumination and depressive symptoms in adults with autism spectrum disorder
Source: PLoS One. 2018 Aug 7;13(8):e0200340. doi: 10.1371/journal.pone.0200340 (PMC6080759; doi:10.1371/journal.pone.0200340)
Supplement: S5 Table — Note: BDI-II = Beck Depression Inventory, 2nd edition; RRS = Ruminative Response Scale; RRS brooding = Ruminative Response Scale, Brooding subscale; RBS-R Total = Repetitive Behavior Scale-Revised overall total score; SRS-RRB = Social Responsiveness Scale, 2nd edition, Restricted Repetitive Behavior subscale T-score; SRS Total = Social Responsiveness Scale, 2nd edition, overall total score; IS = Interests Scale overall “Intensity” score. Bold type indicates significance at p < .05. (DOCX) [file pone.0200340.s009.docx]

*S5 Table. Correlations between demographic and psychometric variables within all typically developing participants (depressed and never-depressed combined)*

| Pearson r | Age | Verbal IQ | Nonverbal | BDI-II | RRS | RRS | RBS-R | SRS-RRB | SRS Total | IS |
| --- | --- | --- | --- | --- | --- | --- | --- | --- | --- | --- |
| p-value |  |  | IQ |  | Total | Brooding |  |  |  | Intensity |
| n |  |  |  |  |  |  |  |  |  |  |
| Age |  |  |  |  |  |  |  |  |  |  |
|  | - |  |  |  |  |  |  |  |  |  |
|  |  |  |  |  |  |  |  |  |  |  |
| Verbal IQ | **0.311** |  |  |  |  |  |  |  |  |  |
|  | 0.018 | - |  |  |  |  |  |  |  |  |
|  | 57 |  |  |  |  |  |  |  |  |  |
| Nonverbal IQ | 0.182 | **0.392** |  |  |  |  |  |  |  |  |
|  | 0.176 | 0.003 | - |  |  |  |  |  |  |  |
|  | 57 | 57 |  |  |  |  |  |  |  |  |
| BDI-II | -0.026 | -0.059 | 0.007 |  |  |  |  |  |  |  |
|  | 0.847 | 0.664 | 0.958 | - |  |  |  |  |  |  |
|  | 56 | 56 | 56 |  |  |  |  |  |  |  |
| RRS Total | -0.003 | -0.034 | -0.091 | **0.780** |  |  |  |  |  |  |
|  | 0.981 | 0.802 | 0.504 | 0.000 | - |  |  |  |  |  |
|  | 56 | 56 | 56 | 56 |  |  |  |  |  |  |
| RRS Brooding | -0.023 | -0.238 | -0.208 | **0.697** | **0.844** |  |  |  |  |  |
|  | 0.865 | 0.077 | 0.123 | 0.000 | 0.000 | - |  |  |  |  |
|  | 56 | 56 | 56 | 56 | 56 |  |  |  |  |  |
| RBS-R | -0.207 | **-0.476** | **-0.468** | **0.437** | **0.364** | **0.508** |  |  |  |  |
|  | 0.137 | 0.000 | 0.000 | 0.001 | 0.007 | 0.000 | - |  |  |  |
|  | 53 | 53 | 53 | 53 | 53 | 53 |  |  |  |  |
| SRS-RRB | -0.122 | **-0.317** | -0.057 | **0.471** | **0.418** | **0.467** | **0.617** |  |  |  |
|  | 0.368 | 0.017 | 0.675 | 0.000 | 0.001 | 0.000 | 0.000 | - |  |  |
|  | 56 | 56 | 56 | 56 | 56 | 56 | 53 |  |  |  |
| SRS Total | -0.133 | **-0.335** | -0.023 | **0.522** | **0.425** | **0.486** | **0.648** | **0.947** |  |  |
|  | 0.329 | 0.012 | 0.867 | 0.000 | 0.001 | 0.000 | 0.000 | 0.000 | - |  |
|  | 56 | 56 | 56 | 56 | 56 | 56 | 53 | 56 |  |  |
| IS Intensity | -0.256 | **-0.303** | -0.198 | **0.458** | **0.418** | **0.500** | **0.682** | **0.624** | **0.694** |  |
|  | 0.064 | 0.027 | 0.156 | 0.001 | 0.002 | 0.000 | 0.000 | 0.000 | 0.000 | - |
|  | 53 | 53 | 53 | 53 | 53 | 53 | 52 | 53 | 53 |  |

*Note*: BDI-II=Beck Depression Inventory, 2^nd^ edition; RRS=Ruminative Response Scale; RRS brooding=Ruminative Response Scale, Brooding subscale; RBS-R Total=Repetitive Behavior Scale-Revised overall total score; SRS-RRB=Social Responsiveness Scale, 2^nd^ edition, Restricted Repetitive Behavior subscale T-score; SRS Total= Social Responsiveness Scale, 2^nd^ edition, overall total score; IS=Interests Scale overall “Intensity” score. Bold type indicates significance at p<.05.
